# Analysis of Metabolite Distribution in Rat Liver of High-Fat Model by Mass Spectrometry Imaging

**DOI:** 10.3390/metabo13030411

**Published:** 2023-03-10

**Authors:** Hongmei Mao, Wenjun Wang, Xuesong Xiang, Yan Li, Jinpeng Zhao, Yin Huang, Shuangshuang Di, Qin Zhuo, Honggang Nie

**Affiliations:** 1 Key Laboratory of Trace Element Nutrition of National Health Commission, National Institute for Nutrition and Health, Chinese Center for Diseases Control and Prevention, Beijing 100050, China; 2 Beijing Junfeix Technology Co., Ltd., Beijing 100081, China; 3 Suzhou PANOMIX Biomedical Technology Co., Ltd., Suzhou 215125, China; 4 Analytical Instrumentation Center, Peking University, Beijing 100871, China; 5 Beijing National Laboratory for Molecular Sciences, College of Chemistry and Molecular Engineering, Peking University, Beijing 100871, China

**Keywords:** MSI, high lipid (HL), rat liver, triglyceride (TG)

## Abstract

Hyperlipidemia is a medical condition characterized by elevated levels of blood lipids, especially triglycerides (TG). However, it remains unclear whether TG levels remain consistently elevated throughout the entire developmental stage of the high-lipid state. In our animal experiment, we found that TG levels were significantly higher in the early stage of the high-lipid model but significantly decreased at the 14th week of the late stage, reaching levels similar to those of the control group. This suggests that TG levels in the high-lipid model are not always higher than those of the control group. To determine the reason for this observation, we used in situ mass spectrometry imaging (MSI) to detect the distribution of metabolites in the liver of rats. The metabolite distribution of the control rats at different stages was significantly different from that of the model rats, and the high-lipid model differed significantly from the control rats. We identified nine functional metabolites that showed differences throughout the period, namely, PA(20:3-OH/i-21:0), PA(20:4-OH/22:6), PG(20:5-OH/i-16:0), PG(22:6-2OH/i-13:0), PG(O-18:0/20:4), PGP(18:3-OH/i-12:0), PGP(PGJ2/i-15:0), SM(d18:0/18:1-2OH), and TG(14:0/14:0/16:0), among which TG was most significantly correlated with hyperlipidemia and high lipid. This study is unique in that it used MSI to reveal the changes in metabolites in situ, showing the distribution of different metabolites or the same metabolite in liver tissue. The findings highlight the importance of considering the animal’s age when using TG as a biomarker for hyperlipidemia. Additionally, the MSI images of the liver in the high-lipid model clearly indicated the distribution and differences of more significant metabolites, providing valuable data for further research into new biomarkers and mechanisms of hyperlipidemia. This new pathway of in situ, visualized, and data-rich metabolomics research provides a more comprehensive understanding of the characteristics of high lipid and its implications for disease prevention and treatment.

## 1. Introduction

Hyperlipidemia is a common metabolic disorder characterized by excessive levels of lipids in the blood, which can lead to serious cardiovascular diseases, diabetes, atherosclerosis, and other related illnesses [1,2,3,4,5]. Hyperlipidemia is often classified into hypercholesterolemia, hypertriglyceridemia, and mixed hyperlipidemia, where both high triglyceride (TG) and high total cholesterol (TC) levels are present simultaneously [6,7]. Numerous studies have demonstrated that a high-lipid diet can cause hyperlipidemia. In fact, the hyperlipidemia rat model induced by a high-lipid diet has been shown to form lesions similar to the early pathological changes seen in humans. These changes are characterized by the deposition of lipid in the liver and dyslipidemia [8]. Therefore, this model can be widely applied in studying the pathogenesis of hyperlipidemia and cardiovascular and cerebrovascular diseases, as well as for screening drugs related to these conditions.

Experimental animals such as rats are commonly used in the study of lipid metabolism due to their strong adaptability, stable key indicators, and good repeatability. During the initial stages of the hyperlipidemia model, hepatocyte steatosis and lipid storage might be closely related to the changes in the content of fatty acids, sterols, and glycerophospholipids [9,10,11]. Overall, hyperlipidemia is a serious metabolic disorder that can have severe consequences if left untreated [12,13]. Fortunately, through the use of animal models such as the hyperlipidemia rat model, we can gain a better understanding of the pathogenesis of this disease and identify potential drug targets for its treatment [14,15].

Lipid enrichment analysis was conducted on differential metabolites found in the serum of hyperlipidemia rat models induced by a high-lipid diet. The analysis revealed significant changes in the contents of seven lipid substances, including fatty acids, sterols, glycerophospholipids, and lipid molecules [10,16,17,18]. Metabolomics offers a novel approach to gaining insight into metabolic profiling and the pathophysiological mechanisms of hyperlipidemia [19]. Current biomarkers, such as triglyceride (TG), total cholesterol (TCHO), low-density lipoprotein cholesterol (LDL-C), high-density lipoprotein cholesterol (HDL-C) [20], and other metabolites [21,22,23,24], lack the necessary information on distribution sites in organs. Therefore, it is crucial to develop techniques capable of in situ imaging and metabolite visualization of organ tissues. Mass spectrometry imaging (MSI), also known as spatial metabolomics, is a promising technique in this regard [25,26,27,28,29]. Although obtaining human tissue samples for studying organ tissues is not convenient, the rat model has been extensively used to investigate human hyperlipidemia. Small animal models have provided significant insights into the investigation of complex multifactorial diseases such as hyperlipidemia [2]. Moreover, animal models have been widely used in metabolomics to study bioactive macromolecules [30] and metabolic pathways related to high-lipid diet-induced obesity [31], as well as in MSI to study steatosis [32]. In summary, lipid enrichment analysis of differential metabolites in the serum of hyperlipidemia rat models induced by high-lipid diets is a useful tool in understanding the underlying mechanisms of this disorder. Metabolomics, particularly MSI, offers exciting opportunities to study metabolic profiling and the pathophysiological mechanisms of hyperlipidemia in organ tissues. Animal models, especially rat models, have been extensively used in these investigations and have provided valuable insights into complex diseases such as hyperlipidemia.

## 2. Materials and Methods

### 2.1. Rat Model and Biochemistry Measurement

A total of 20 SPF-grade male SD rats, weighing 180–200 g, were purchased from Beijing Weitonglihua Experimental Animal Technology Co., Ltd. (Beijing, China) After adaptive feeding for 7 days, the weight and contents of TG in blood were measured. After the abnormalities were excluded, they were randomly divided into the control group (control) and the model group (high lipid, HL). The control group was given a basic diet, and the model group was given a high-fat diet. After 2 weeks of giving the corresponding diet, blood was taken from the inner canthus of all animals to detect serum TG. After successful modeling, rats were fed continuously for 12 weeks and then killed.

The formula feed of the mixed hyperlipidemia animal model was added with 20.0% sucrose, 15% lard oil, 1.2% cholesterol, 0.2% sodium cholate, 1.2% calcium hydrogen phosphate, 0.8% stone powder, and 5.0% casein in the maintenance feed to prepare the high-fat feed. All the feed was provided by Beijing Keaoxieli Feed Co., Ltd. (Beijing, China), with the production license No. of SCXK (Beijing) 2014-0010.

The blood lipid test kit (TCHO/TG/LDL-c/HDL-c) was purchased from Wako Company, Japan (data in Figure S1).

### 2.2. Liver Tissues Stained with Hematoxylin and Eosin (H&E)

Some fragments of the liver of each animal were instantly fixed [freshly prepared formaldehyde 4% (wt./vol.) in 0.1 M phosphate buffer, pH 7.2] for 48 h at room temperature. Random fragments were dehydrated in graded alcohols of increasing concentration to absolute alcohol and were then cleared in xylene and embedded in Paraplast Plus (Sigma-Aldrich, St. Louis, MO, USA). The material was sectioned at 5 μm and then stained with H&E.

### 2.3. Mass Spectrometry Imaging (MSI)

The liver of different rat in slices of 10 μm thickness was prepared using a freezing microtome (CM1950; Leica, Nussloch, Germany) and mounted on the ITO-coated glass slides for MALDI-MSI. The matrix 2,5-dihydroxybenzoic acid (DHB) was sublimated using the iMLayer vacuum deposition system (Shimadzu, Kyoto, Japan). DHB crystals formed on the specimen surface at a thickness of 1.5 μm. The tissue sections were analyzed using a Shimadzu iMScope TRIO (Shimadzu, Japan). Optical images of sections were acquired using iMScope before all samples were prepared. The laser-irradiated spot was 200 shots, and the repetition rate was 1000 Hz. The sample slice was scanned with the resolution ratio of 50 μm × 50 μm under positive and negative ion modes. The MS data were acquired in the mass range of *m*/*z* 400–900, and laser power was 60% (Shimadzu’s notation), while the sample voltage was applied at 3.5 and 3.0 kV. The MSI data were analyzed using the software of Imaging MS solution Ver.1.30, focusing on the regions manually defined according to both the optical image and MSI data image, which can be used for the comparison of the difference in the livers of different rats.

### 2.4. Data Processing and Drawing

The ion peaks detected were searched in eight databases, namely, KEGG, BioCyc, HMDB, DrugBank, LipidMaps, ChEBI, PubChem, and MoNA, to annotate primary metabolites. Metabolite co-expression calculation analysis and metabolic network enrichment annotation were performed on the identified metabolite list using the Mummichog method to uncover the functions of metabolite molecules [33]. The pretreated data were analyzed using the UMAP Manifold method combined with the data analysis method to conduct spatial automatic segmentation analysis and obtain the optimal metabolite cluster and metabolite characteristics [34,35,36,37,38]. This generated single-cell metabolism clustering result data that could distinguish between different cell spatial heterogeneity. On the basis of the obtained spatial classification region results, large-scale bootstrap random sampling was performed to obtain large-queue spatial sample expression data. Large cohort data were used to identify specific differential metabolites between spatial subtypes, and biological mechanisms were explained on the basis of KEGG metabolic pathway analysis.

To calculate the difference *p*-value, a *t*-test was performed, and the histogram was plotted using GraphPad Prism V8.8 and R language. The data were expressed as mean ± SE. Metabolites that met the following conditions were considered differential metabolites: fold change >1.5 or <0.75, *p* < 0.05, VIP >1. Otherwise, they were considered non-differential metabolites.

## 3. Results

### 3.1. Pathological Results of Control and High-Lipid Models (HL)

**Figure 1:**
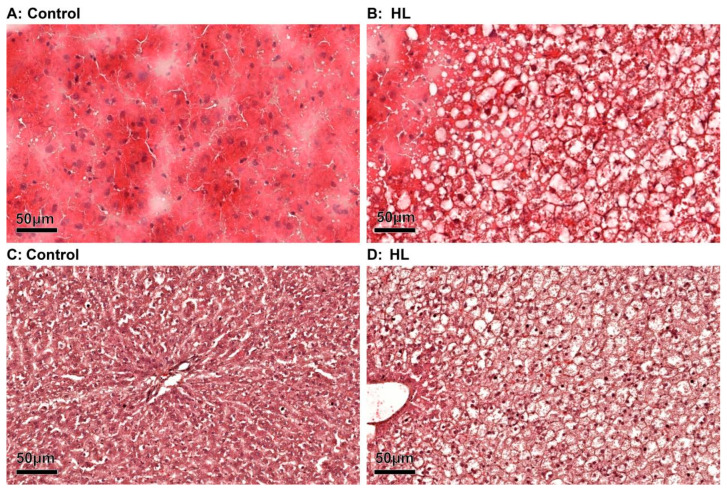
Observation of fat deposition in liver tissues under a light microscope (H&E staining ×200). (**A**) H&E staining from frozen sections in control of rat liver; (**B**) H&E staining from frozen sections in HL of rat liver; (**C**) H&E staining from paraffin sections in control of rat liver; (**D**) H&E staining from paraffin sections in HL of rat liver. The scale bar is 50 μm; *n* = 3; the sample repeat for each group was three rats.

We conducted HE staining on both frozen sections (Figure 1A,B) and paraffin sections (Figure 1C,D) to analyze the pathological states of rat liver. In the control group (Figure 1A,C), rat hepatocytes showed regular and radial arrangement with uniform size. The structure and morphology of the liver were normal. In contrast, in the model group (Figure 1B,D), the hepatocytes appeared morphologically swollen, and a large number of fat vacuoles were visible. The liver showed diffuse steatosis and fuzzy intercellular boundary. These observations indicate significant liver damage due to hyperlipidemia.

### 3.2. Changes of Body Weight and Blood TG Content with Time in Rats

**Figure 2:**
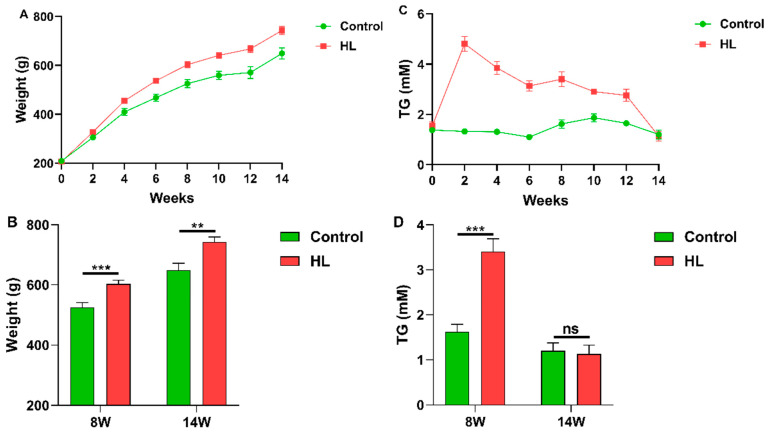
Weight and blood TG in the rat. (**A**) Change in weight over time. (**B**) The weight difference between the control and HL groups at 8 W (8 weeks) and 14 W (14 weeks). (**C**) Change in blood TG with time. (**D**) The blood TG difference between control and HL groups at 8 W (8 weeks) and 14 W (14 weeks). ** *p* < 0.01; *** *p* < 0.001; ns, no significance. Green lines and columns denote the control; red lines and columns denote the HL. *n* = 8; the sample repeat for each group was eight rats.

The key factor in high lipid levels is the blood TG content. In order to investigate this, the TG content in rat blood was continuously measured over time, as shown in Figure 2C. The results indicated that the TG content in the hyperlipidemia (HL) group was significantly higher than that in the control group (*p* < 0.001), as shown in Figure 2D. However, there was no significant difference between the TG contents of the two groups at 14 weeks (*p* > 0.05) (Figure 2D). It was also observed that the body weights of rats in the control and HL groups differed significantly, with the HL group having a significantly higher body weight, as shown in Figure 2A,B.

### 3.3. Photo of MSI and Mass Spectrum in Liver Tissues

**Figure 3:**
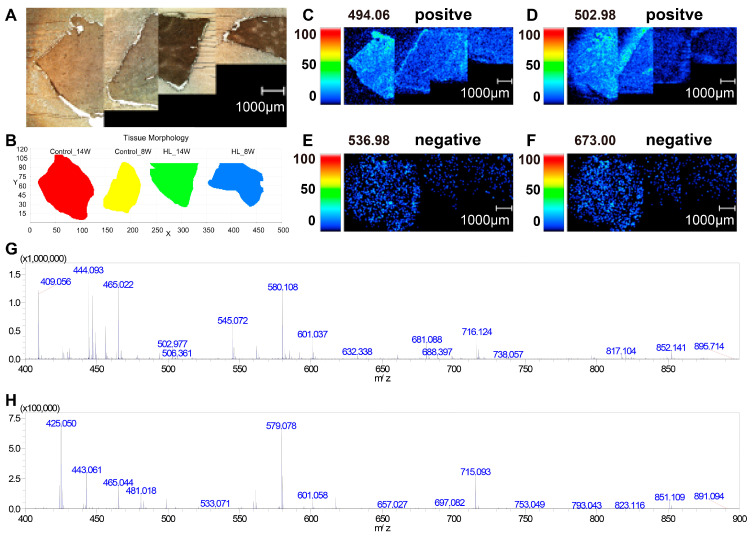
MSI intensity images for various *m*/*z* values and mass spectrum in liver tissues. (**A**) The optical images from left to right are Control_14w (control 14 weeks), Control_8w (control 8 weeks), HL_14w (high-lipid 14 weeks), and HL_8w (high-lipid 8 weeks). (**B**) The simulation diagram of the sample; the sequence of samples from left to right is the same as that in (**A**). (**C**) The representative MSI of positive ion mode was *m*/*z* 494.06; the sequence of samples from left to right is the same as that in (**A**). (**D**) The representative MSI of positive ion mode was *m*/*z* 502.98; the sequence of samples from left to right is the same as that in (**A**). (**E**) The representative MSI of negative ion mode was *m*/*z* 536.98; the sequence of samples from left to right is the same as that in (**A**). (**F**) The representative MSI of negative ion mode was *m*/*z* 673.00; the sequence of samples from left to right is the same as that in (**A**). (**G**) Mass spectrum in positive ion mode. (**H**) Mass spectrum in negative ion mode. The bar scale is 1000 μm.

Frozen liver sections from four groups (Control_14w, Control_8w, HL_14w, and HL_8w) were mounted on ITO slides before sublimating the matrix. Optical images (Figure 3A) were obtained before normalizing the picture to coordinates, resulting in a simulated image (Figure 3B). Figure 3C–F show representative mass intensity images for various *m*/*z* values in positive and negative ion modes. The mass spectra of the four groups in positive and negative ion modes are displayed in Figure 3G and Figure 3H, respectively. The results indicate that mass spectral imaging images were remarkably different after the four groups of samples were fixed on one slide and simultaneously scanned, and the mass spectral peaks were mainly distributed within the range of *m*/*z* 400–750.

### 3.4. PLS-DA Analysis

**Figure 4:**
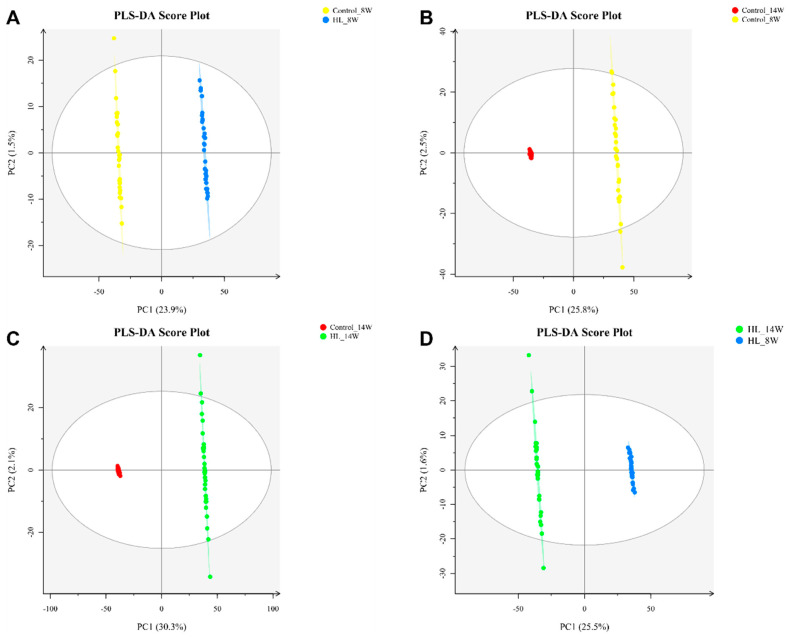
PLS-DA scores of four comparisons in liver tissues. (**A**) PLS-DA score of Control_8W vs. HL_8W. (**B**) PLS-DA score of Control_14W vs. Control_8W. (**C**) PLS-DA score of Control_14W vs. HL_14W. (**D**) PLS-DA score of HL_14W vs. HL_8W. *n* = 4; the sample repeat for each group was four rats.

PLS-DA analysis (Figure 4) was conducted on all *m*/*z* values in each sample, and comparisons were made among the four groups: Control_8W vs. HL_8W (Figure 4A), Control_14W vs. Control_8W (Figure 4B), Control_14W vs. HL_14W (Figure 4C), and HL_14W vs. HL_8W (Figure 4D). The results revealed that each comparison could effectively distinguish between the two groups, suggesting that rat metabolism varied at different timepoints and that the metabolites of the control and high-lipid model groups were significantly distinct.

### 3.5. Differential Metabolites

**Figure 5:**
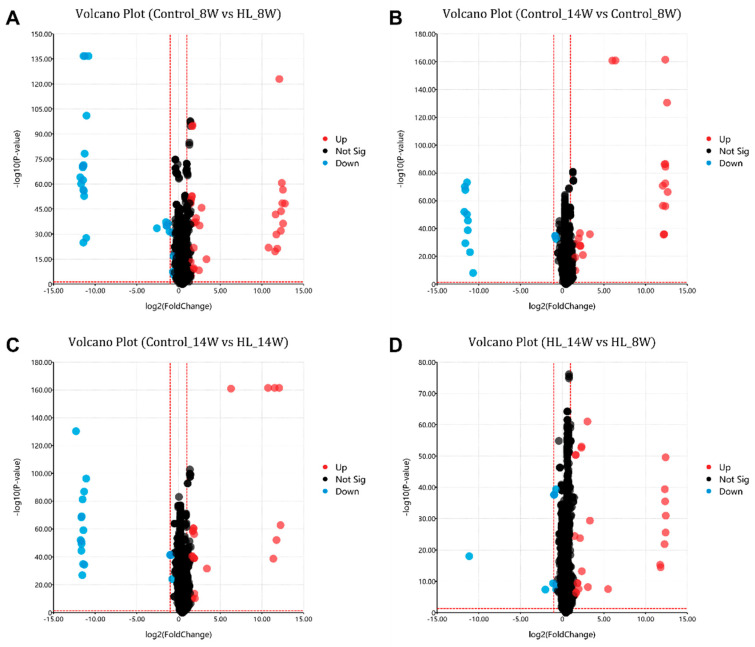
Volcanic diagram analysis of differential metabolites. (**A**) Volcanic diagram analysis of Control_8W vs. HL_8W. (**B**) Volcanic diagram analysis of Control_14W vs. Control_8W. (**C**) Volcanic diagram analysis of Control_14W vs. HL_14W. (**D**) Volcanic diagram analysis of HL_14W vs. HL_8W. Red, blue, and black points denote upregulated metabolites, downregulated metabolites, and nonsignificant metabolites, respectively. *n* = 4; the sample repeat for each group was four rats.

After analyzing the volcano plot (Figure 5), metabolites with a fold change >2, *p*-value <0.01, and VIP score >1 were selected. In the comparison of Control_8W vs. HL_8W (C1), 37 metabolites were found to be increased and 27 were found to be decreased. In the comparison of Control_14W vs. Control_8W (C2), 25 metabolites were found to be increased and 13 were found to be decreased. In the comparison of Control_14W vs. HL_14W (C3), 25 metabolites were found to be increased and 18 were found to be decreased. In the comparison of HL_14W vs. HL_8W (C4), 27 metabolites were found to be increased and nine were found to be decreased. These results suggest that the HL group had more increased metabolites and fewer decreased ones, possibly due to the accumulation of metabolites caused by high lipids in the rat liver.

### 3.6. MSI of Differential Metabolites

**Figure 6:**
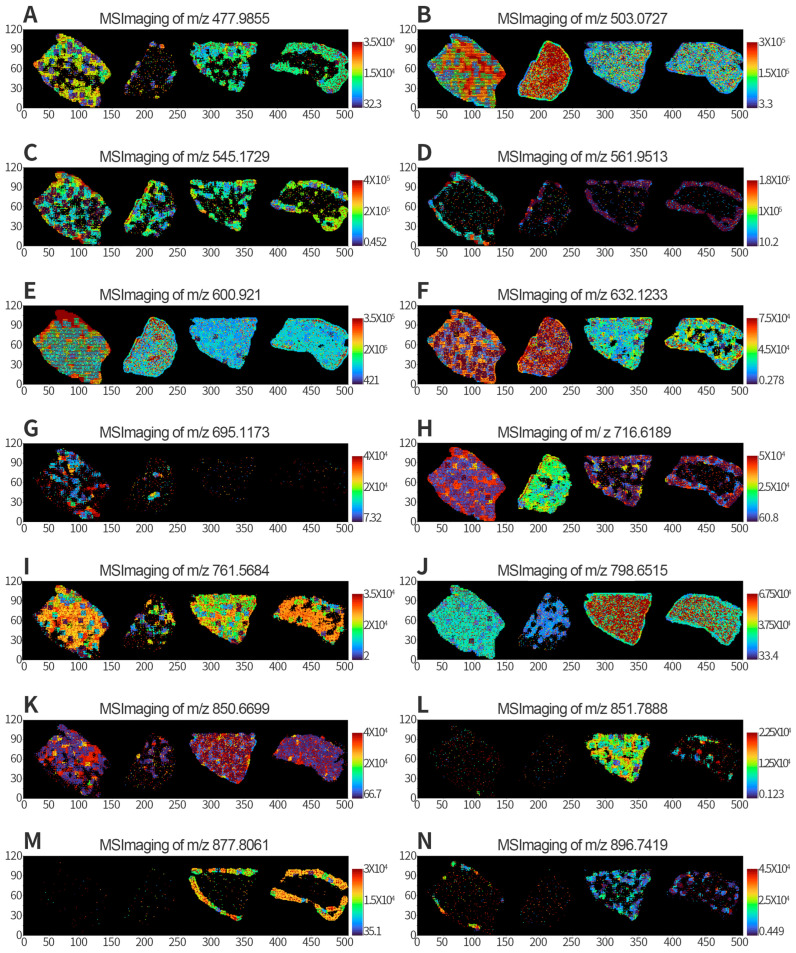
MSI of differential metabolites from different samples in rat liver. (**A**–**N**) Metabolite intensity images for various *m*/*z* of 477.9855, 503.0727, 545.1729, 561.9513, 600.921, 632.1233, 695.1173, 716.6189, 761.5684, 798.6515, 850.6699, 851.7888, 877.8061, and 896.7419, respectively.

Comparative analysis was performed on substances with different *m*/*z* values, and 14 metabolites were identified in key comparisons C2 and C3. MSI images of these 14 metabolites were extracted and are shown in Figure 6. These differential metabolites were briefly described, with the two key ones being *m*/*z* 716.6189 (Figure 6H) and *m*/*z* 850.6699 (Figure 6K). The MSI results clearly showed significant differences in the distribution of metabolites before and after high-lipid treatment, and the differences were present at different growth stages.

### 3.7. Correlation Analysis between TG and Differential Metabolites

**Figure 7:**
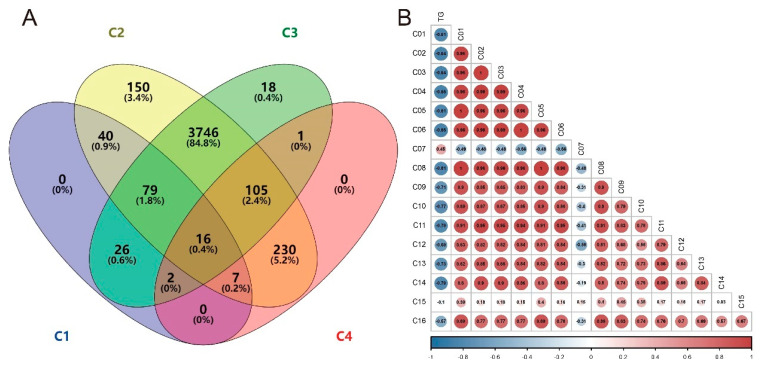
Venn diagram of four comparisons in differential metabolites and correlation diagram. (**A**) Venn diagram of four comparisons. C1, C2, C3, and C4 are Control_8W vs. HL_8W, Control_14W vs. Control_8W, Control_14W vs. HL_14W, and HL_14W vs. HL_8W, respectively. (**B**) Correlation analysis of TG and 16 different metabolites. Blue circles are negative correlations, while red circles are positive correlations. The number in the circle represents the correlation coefficient. A correlation coefficient >0.6 represents a positive, while a correlation coefficient <−0.6 represents a negative.

We screened for differential metabolites according to the change trend of TG, where differences existed between Control_8W and HL_8W (C1), no differences existed between Control_14W and Control_8W (C2), no differences existed between Control_14W and HL_14W (C3), and differences existed between HL_14W and HL_8W (C4). The metabolites common to all comparisons are shown in the Venn diagram (Figure 7A), with 16 metabolites identified and listed in Supplementary Table S1. Nine of these metabolites belonged to four categories: phosphatidic acids (PA), phosphatidylglycerols (PG), Sphingomyelins (SM), and triacylglycerols (TG). Correlations between TG and the differential 16 metabolites were analyzed, with Figure 7B showing a negative correlation between TG and most differential metabolites, with a correlation coefficient greater than 0.6 (correlation coefficient of negative correlation less than −0.6). The identified metabolites were C01 [PA(20:3-OH/i-21:0)], C02 [PA(20:4-OH/22:6)], C03 [PG(20:5-OH/i-16:0)], C04 [PG(22:6-2OH/i-13:0)], C05 [PG(O-18:0/20:4)], C06 [PGP(18:3-OH/i-12:0)], C07 [PGP(PGJ2/i-15:0)], C08 [SM(d18:0/18:1-2OH)], and C09 [TG(14:0/14:0/16:0)]. This result indicated that TG was mainly negatively correlated with the differential metabolites, and the accumulation of TG in the high-lipid state would lead to a significant decline in other lipids.

## 4. Discussion

Hyperlipidemia is a growing public health concern attributed to the increased consumption of fatty foods. To study the effects of high-lipid diets on animal models, we established a high-lipid animal model by feeding rats with a high-lipid diet. At the early stages of model establishment, we observed a significant increase in triglyceride (TG) levels, and the liver pathology results showed an increase in TCHO, LDL, cell size, and body weight.

TG is a critical lipid molecule involved in energy storage and metabolism [39,40]. Hyperlipidemia, a condition characterized by abnormally high levels of lipids in the blood, particularly TG and cholesterol, is closely related to a high-lipid diet and obesity [41,42]. Excessive intake of dietary fat can lead to the accumulation of TG in adipose tissue and liver, causing hepatic steatosis and metabolic disorders such as insulin resistance and type 2 diabetes [2,42,43,44]. TG had a significant increase in the high-lipid model, but TG had a downward trend with the growth time, and it was the same as the control (there was no difference) at the 14th week. This special change may indicate different phenomena. The TCHO, HDL, and LDL (Supplementary Figure S1) showed significant changes at 10 W, but this change trend was different from TG. The specific reason is unknown, and more discussion and analysis are required. We do not discuss this in this article; it will be verified in future work. We analyzed the changes in metabolites through MSI; the metabolites changed significantly at different times, and the metabolites in the high-lipid model also changed significantly. It can be clearly seen in the charts analyzed by PLS-DA model scores that the metabolism of rats at different stages was also significantly different, the metabolites of high-lipid model rats and control rats were also significantly different, and the increased metabolites were more (volcanic diagram in Figure 5). We extracted and displayed the MSI images of differential metabolites (Figure 6) and found that the content and distribution of these metabolites were significantly different in the four groups. This difference is different from previous metabolomics of tissue or serum, in that it can reveal the changes of metabolites in situ and show the distribution status of different metabolites or the same metabolite in liver tissue. Lastly, through Venn and correlation analysis, we found that the main changed metabolites were probably TG (14:0/14:0/16:0), which were closely related to high fat or hyperlipidemia and could reflect the occurrence of hyperlipidemia. Egg yolk (EY) can alleviate the hyperlipidemia in HFD-induced obese mice by increasing serum phosphatidic acids (PA), phosphatidylglycerols (PG), sphingomyelins (SM), and triglycerides (TG) which are closely associated with glycerophospholipid metabolism [45]. This provides further evidence of the potential health benefits of EY consumption in reducing hyperlipidemia and its associated metabolic disorders. Our findings suggest that TG (14:0/14:0/16:0) is closely related to hyperlipidemia induced by a high-lipid diet. This is consistent with previous studies that have shown that increased levels of TG are a hallmark of hyperlipidemia [46]. Furthermore, the decrease in TG levels observed over time in our study may be due to the adaptation of the liver to the high-lipid diet or to the natural regulation of lipid metabolism in the liver.

Our study revealed that high-lipid diets can induce hyperlipidemia in rats, which is characterized by elevated levels of triglycerides (TG) and cholesterol. Our findings suggest that the increase in TG levels is closely related to the development of hyperlipidemia induced by a high-lipid diet, consistent with previous studies. Through MSI analysis, we identified differential metabolites in liver tissue and found that the changes in metabolites were time-dependent. The volcanic diagram and Venn and correlation analysis revealed that TG (14:0/14:0/16:0) may play a key role in the development of hyperlipidemia. Interestingly, we observed a downward trend in TG levels over time, which could be due to the liver’s adaptation to the high-lipid diet or natural regulation of lipid metabolism. Our study highlights the potential of MSI as a powerful tool for in situ metabolite analysis in liver tissue, providing more comprehensive and accurate information than traditional metabolomics studies. Overall, our results provide new insights into the underlying mechanisms of hyperlipidemia and suggest potential strategies for its prevention and treatment. Further research is needed to confirm these findings and explore the clinical applications of MSI in the diagnosis and treatment of hyperlipidemia and related metabolic disorders.

## 5. Conclusions

The results of our study have significant implications for understanding the development and progression of hyperlipidemia. Our study used a high-lipid animal model by feeding rats with a high-lipid diet, which is a relevant model for understanding the mechanisms underlying the development of hyperlipidemia. By monitoring TG levels in the model, we found that the initial increase in TG levels was followed by a decrease, a novel finding that may have important implications for the use of TG as a biomarker of hyperlipidemia. We used MSI in this study to examine the distribution and differences of metabolites in different tissues and different sites of the same tissue, revealing changes that would not have been detected using previous metabolomics methods. Our results identified TG (14:0/14:0/16:0) as a main metabolite closely related to high fat and hyperlipidemia, reflecting the occurrence of hyperlipidemia. This finding provides new insights into the metabolic changes that occur in hyperlipidemia and may help to identify new therapeutic targets for the condition. In conclusion, our study provides important implications for understanding the mechanisms underlying hyperlipidemia and for the development of new treatments for this condition. The use of MSI in this study demonstrated its potential as a powerful tool for the study of metabolomics and the identification of biomarkers in situ. These results provide a valuable basis for future research into the pathophysiology of hyperlipidemia and the identification of new therapeutic targets for this condition.

## Data Availability

The data presented in this study are available in the main article and the Supplementary Materials.

## Supplementary Materials

The following supporting information can be downloaded at: https://www.mdpi.com/article/10.3390/metabo13030411/s1, Figure S1: TCHO, HDL and LDL content in rat serum. Green lines and columns are present Control, red lines and columns are present HL; Table S1: 16 identified metabolites in the Venn diagram (Figure 7A).

## Abbreviations

HDL-C	High-density lipoprotein cholesterol
H&E	Hematoxylin and eosin
HFD	High-fat diet
HL	High-lipid or high-fat
LDL-C	Low-density lipoprotein cholesterol
MSI	Mass spectrometry imaging
PA	Phosphatidic acids
PG	Phosphatidylglycerols
PLS-DA	Partial least squares discriminant analysis
SE	Standard error
SM	Sphingomyelins
SPF	Specific pathogen-free
TC	Total cholesterol
TG	Triglyceride
VIP	Variable importance in the projection
