# Supplementary material for: Analysis of Metabolite Distribution in Rat Liver of High-Fat Model by Mass Spectrometry Imaging
Source: Metabolites. 2023 Mar 10;13(3):411. doi: 10.3390/metabo13030411 (PMC10057431; doi:10.3390/metabo13030411)
Supplement: Supplementary file 1 [file metabolites-13-00411-s001.zip › metabolites-2216586-supplementary.pdf]

Analysis of Metabolite Distribution in Rat Liver of High-Fat Model by Mass Spectrometry Imaging

Hongmei Mao[1], Wenjun Wang[2], Xuesong Xiang[1], Yan Li[1], Jinpeng Zhao[1], Yin Huang[3], Shuangshuang Di[4,5], Qin Zhuo[1*] and Honggang Nie[4,5*]

1 Key Laboratory of Trace Element Nutrition of National Health Commission, National Institute for Nutrition and Health, Chinese Center for Diseases Control and Prevention, Beijing 100050, China
2 Beijing Junfeix Technology Co., Ltd, Beijing 100081, China
3 Suzhou PANOMIX Biomedical Technology Co., Ltd, Suzhou 215125, China
4 Analytical Instrumentation Center, Peking University, Beijing 100871, China
5 Beijing National Laboratory for Molecular Sciences, College of Chemistry and Molecular Engineering, Peking University, Beijing 100871, China
* Correspondence: zhuoqin@ninh.chinacdc.cn (Q.Z.); hgnie@pku.edu.cn (H.N.)

Supplementary Materials:

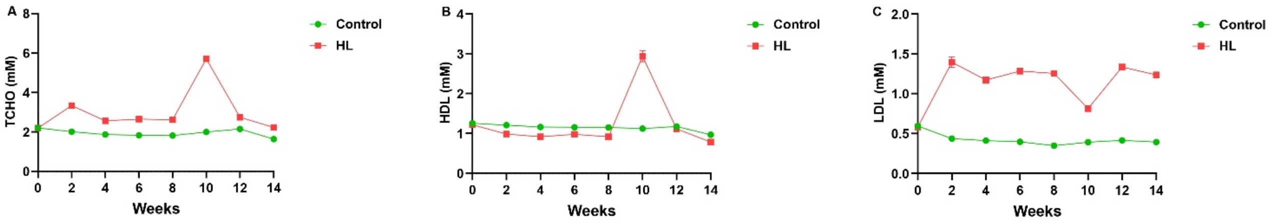

Figure S1: TCHO, HDL and LDL content in rat serum. Green lines and columns are present Control, red lines and columns are present HL.

Table S1 16 identified metabolites in the Venn diagram (Figure 7A)

Table S1-1

No.	MS number	m/z	Identification	Adduct	Formula	Exact_mass	Hmdb/lipidmaps
C01	MSI03462	785.5567	PA(20:3-OH/i-21:0)	[M+H]+	C44H81O9P	762.5774	HMDB0268345
C02	MSI02408	785.4830	PA(20:4-OH/22:6)	[M+H]+	C43H70O11	784.4679	HMDB0266196
C03	MSI04736	785.5004	PG(20:5-OH/i-16:0)	[M+H]+	C42H73O11P	784.4891	HMDB0271531
C04	MSI00846	785.4549	PG(22:6-2OH/i-13:0)	[M+H]+	C41H69O12P	784.4526	HMDB0271238
C05	MSI00714	785.5701	PG(O-18:0/20:4)	[M+H]+	C44H81O9P	784.5618	LMGP04020071
C06	MSI01398	785.4075	PGP(18:3-OH/i-12:0)	[M+H]+	C36H66O14P2	784.3928	HMDB0274876
C07	MSI03632	867.4358	PGP(PGJ2/i-15:0)	[M+H]+	C41H72O15P2	866.4346	HMDB0275130
C08	MSI01257	785.5775	SM(d18:0/18:1-2OH)	[M+Na]+	C41H83N2O8P	762.5887	HMDB0290479
C09	MSI03021	768.6707	TG(14:0/14:0/16:0)	[M+NH4]+	C47H90O6	750.6737	HMDB0042063
C10	MSI04969	803.2670	not identified				
C11	MSI00950	549.4876	not identified				
C12	MSI02187	696.9602	not identified				
C13	MSI04057	659.1703	not identified				
C14	MSI03088	857.0959	not identified				
C15	MSI00577	898.7447	not identified				
C16	MSI04181	665.0880	not identified				

Table S1-2

	C1-Control_8W vs HL_8W			C2-Control_14W vs Control_8W			C3-Control_14W vs HL_14W			C4-HL_14W vs HL_8W		
No.	Fold	p value	VIP	Fold	p value	VIP	Fold	p value	VIP	Fold	p value	VIP
C01	0.5265	0.0000	1.4227	1.0175	0.0153	0.7678	0.9117	0.0000	1.0722	1.7620	0.0000	1.2787
C02	0.5355	0.0000	1.4139	1.0933	0.0000	1.1046	0.9866	0.0069	0.7498	2.0142	0.0000	1.2844
C03	0.5355	0.0000	1.4139	1.0933	0.0000	1.1046	0.9866	0.0069	0.7498	2.0142	0.0000	1.2844
C04	0.3389	0.0000	1.4111	1.0405	0.0986	0.5526	1.0433	0.0007	0.8795	3.2032	0.0000	1.2822
C05	0.5621	0.0000	1.4215	1.0171	0.0214	0.7354	0.9140	0.0000	1.0699	1.6537	0.0000	1.2769
C06	0.3322	0.0000	1.4144	1.0365	0.0818	0.5790	1.0380	0.0011	0.8581	3.2390	0.0000	1.2823
C07	1.7009	0.0000	1.3769	1.6633	0.0000	0.5037	0.6539	0.0000	0.5273	0.6395	0.0000	1.1877
C08	0.5623	0.0000	1.4215	1.0169	0.0220	0.7328	0.9144	0.0000	1.0695	1.6535	0.0000	1.2769
C09	0.5589	0.0000	1.3832	1.0722	0.0010	0.9598	0.8398	0.0129	0.7037	1.6113	0.0001	1.0486
C10	0.5583	0.0000	1.2497	1.0522	0.0184	0.7503	0.8917	0.0007	0.8779	1.6804	0.0001	1.0723
C11	0.5993	0.0000	1.2978	1.1395	0.0014	0.9390	0.9653	0.0001	0.9720	1.8353	0.0000	1.2457
C12	0.4208	0.0001	1.1778	0.8958	0.0004	1.0062	1.0866	0.0086	0.7345	2.3130	0.0001	1.0438
C13	0.6823	0.0009	1.0664	1.1653	0.0005	0.9951	0.9523	0.0013	0.8471	1.6266	0.0000	1.1079
C14	0.2627	0.0000	1.3010	1.8486	0.0000	0.9828	0.9241	0.0329	0.7861	6.5020	0.0000	1.2672
C15	0.6508	0.0000	1.4177	0.9996	0.8894	0.8071	0.3854	0.0000	0.9866	0.5920	0.0000	1.2656
C16	0.4033	0.0000	1.3874	0.9741	0.0605	1.1502	0.6966	0.0000	0.9367	1.6823	0.0002	1.0323
